# Supplementary material for: Sea level rise from West Antarctic mass loss significantly modified by large snowfall anomalies
Source: Nat Commun. 2023 Mar 17;14:1479. doi: 10.1038/s41467-023-36990-3 (PMC10023770; doi:10.1038/s41467-023-36990-3)
Supplement: Supplementary file 1 — Supplementary Information [file 41467_2023_36990_MOESM1_ESM.pdf]

Supplementary Information for: Sea level rise from West Antarctic mass loss significantly modified by large snowfall anomalies

Benjamin J. Davison[1,*], Anna E. Hogg[1], Richard Rigby[1], Sanne Veldhuijsen[2], Jan Melchior van Wessem[2], Michiel R. van den Broeke[2], Paul R. Holland[3], Heather L. Selley[1], Pierre Dutrieux[3]

[1]School of Earth and Environment, University of Leeds, UK

[2]Institute for Marine and Atmospheric Research Utrecht, Utrecht University, the Netherlands

[3]British Antarctic Survey, Cambridge, UK

***corresponding author: b.davison@leeds.ac.uk**

Summary

This document contains auxillary figures that support the results presented in the main text. Figures are provided in the order that they are cited in the main text.

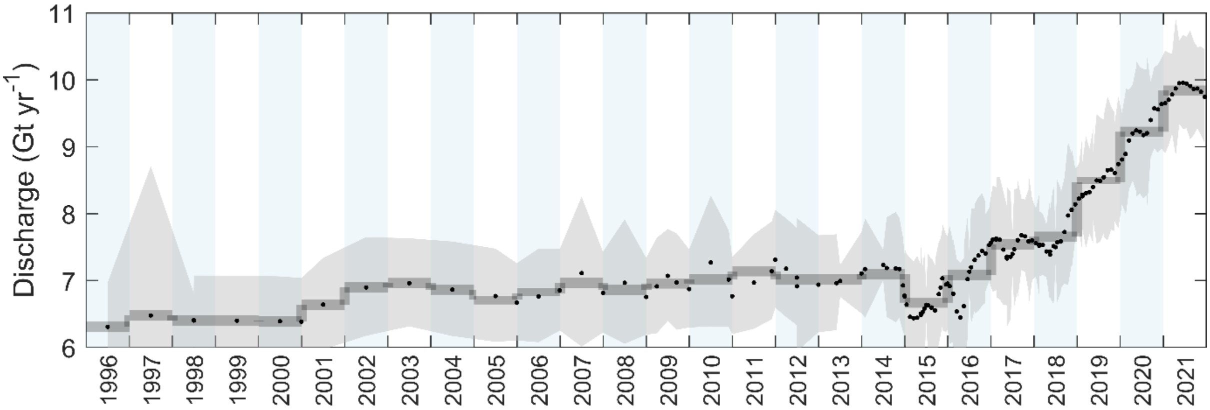

Supplementary Figure 1. Discharge from the Pine Island Glacier southwest tributary, named Piglet Glacier, showing individual discharge observations (black dots) and their errors (vertical grey lines), along with the annual averages (horizontal grey bars). No predefined basin for this glacier exists, so a segment of Pine Island Glacier flux gate was manually selected for this analysis.

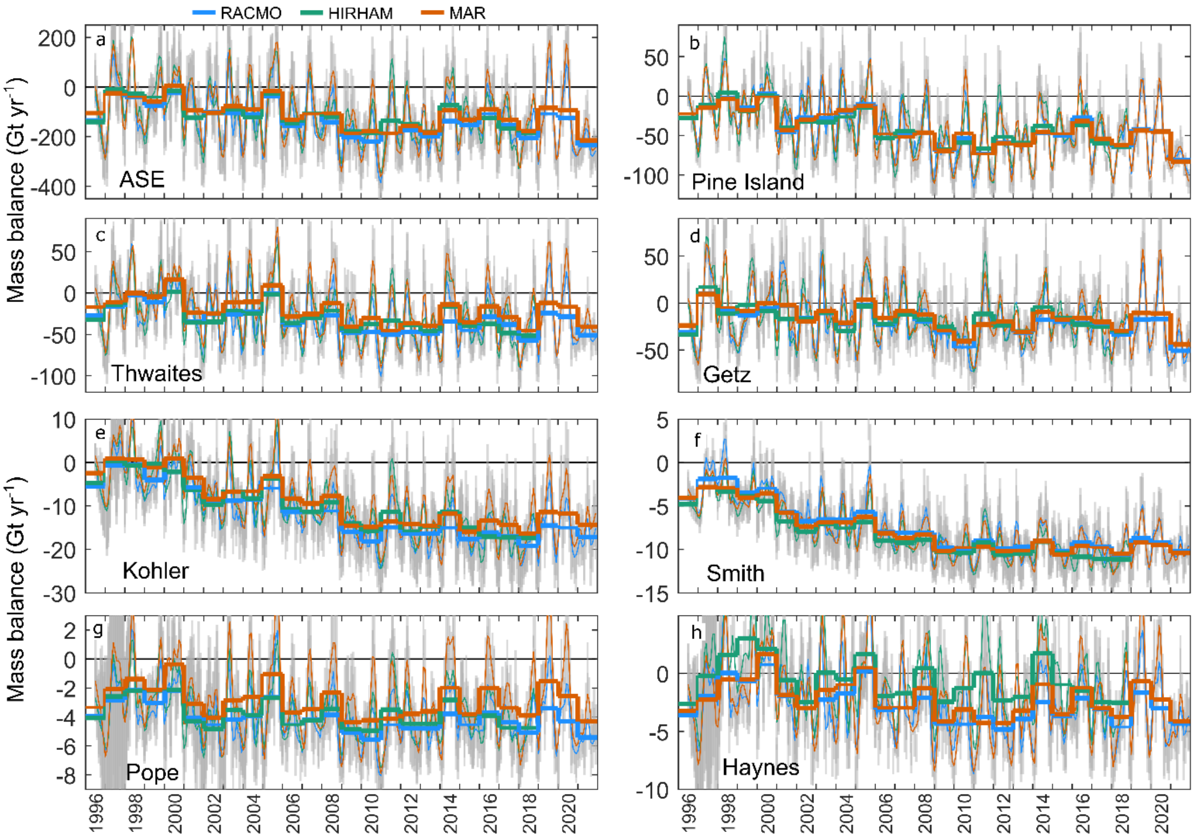

Supplementary Figure 2. Sensitivity of the input-output mass balance estimate to the choice of regional climate model used. (a-f) The annual mass balance (thick coloured lines) and monthly mass balance smoothed with a 3-month moving window (thin coloured lines) and the errors on individual mass balance observations (vertical grey lines) for each regional climate model.

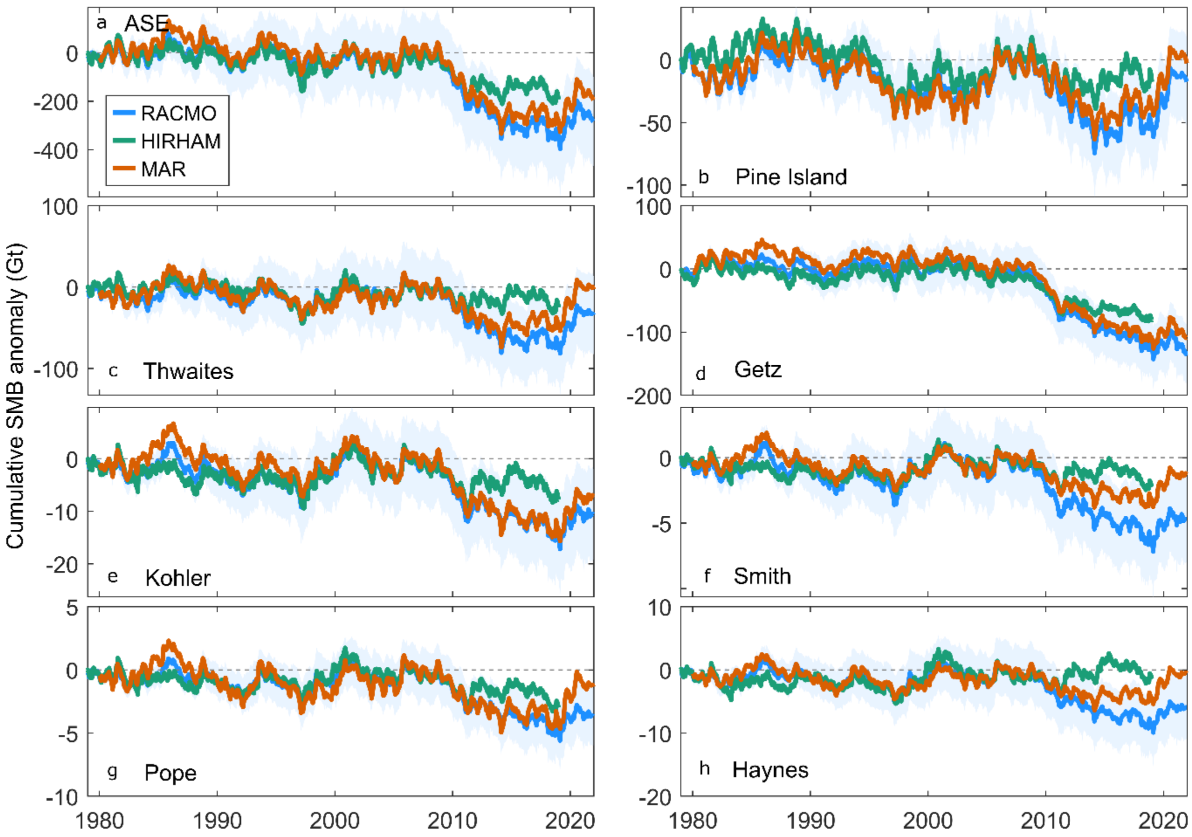

Supplementary Figure 3. (a-f) Cumulative anomalies in surface mass balance relative to the 1980-2008 climatological mean in each basin[1,2] for each of RACMO (blue), HIRHAM (green) and MAR (orange).

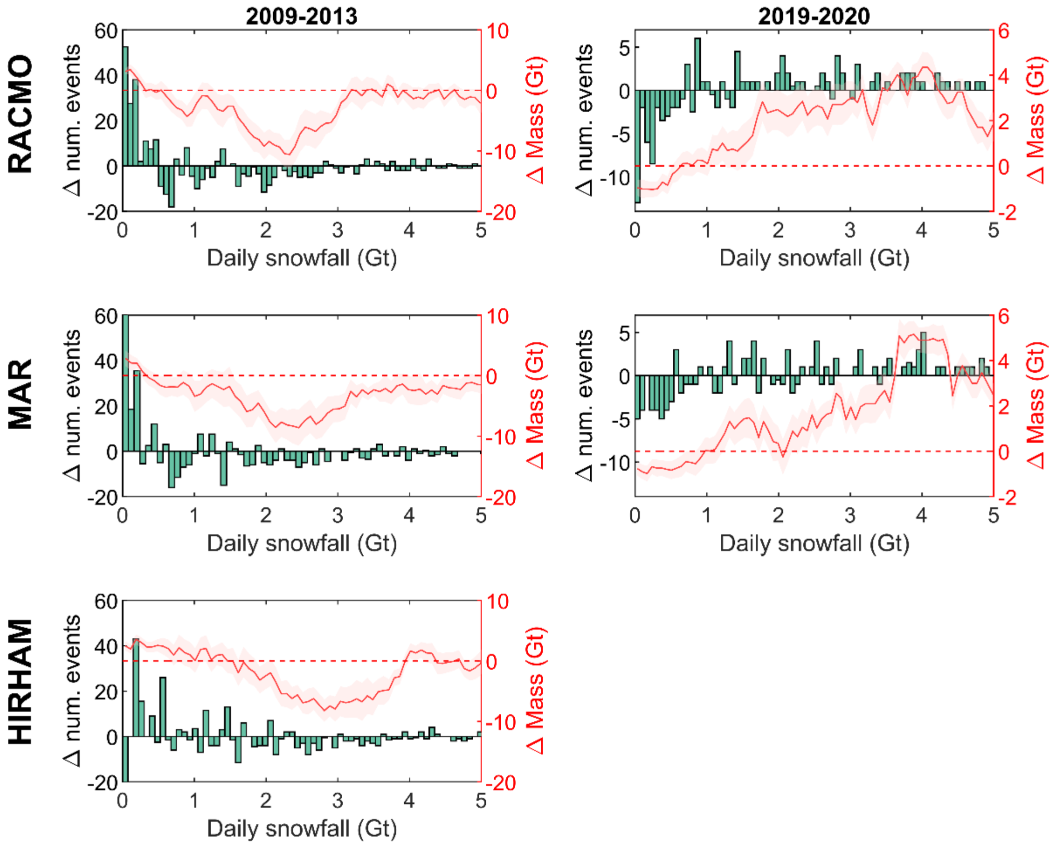

Supplementary Figure 4. Histograms of the change in the number of daily snowfall event sizes (green) and associated mass changes (red) during 2009-2013 compared to all other 5-year periods from 1980-2018 (left column) and the 2019 and 2020 winters (JJA) compared to all other consecutive winters during 1980-2018 (right column). Rows correspond to each regional climate model and all plots show the Amundsen Sea Embayment total. Note that HIRHAM is only available up to 2018.

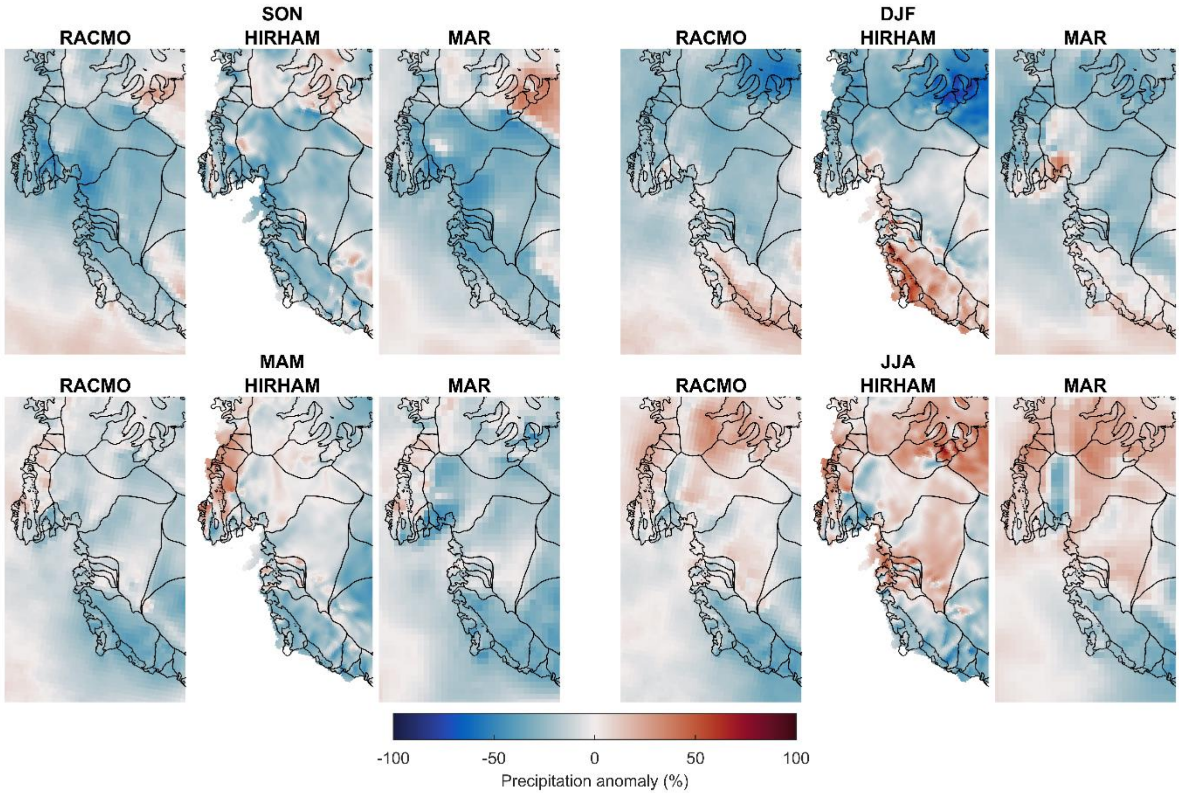

Supplementary Figure 5. Seasonal 2009-2013 precipitation anomalies relative to the seasonal 1980-2008 climatological mean in each of RACMO, HIRHAM and MAR, with the Antarctic Ice Sheet coastline (black line) also shown[1,2].

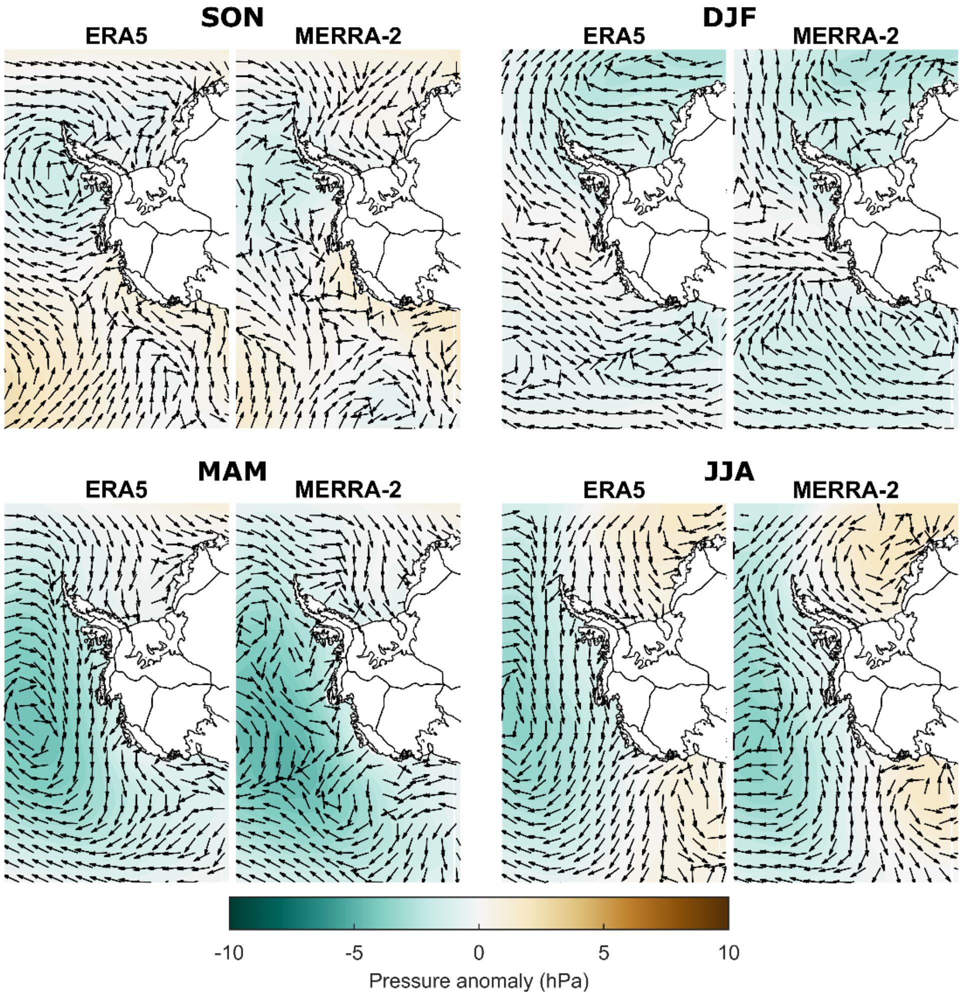

Supplementary Figure 6. Seasonal 2009-2013 surface pressure and wind direction anomalies relative to the seasonal 1980-2008 climatological mean in each of ERA5 and MERRA-2, with the Antarctic Ice Sheet coastline (black line) also shown[1,2].

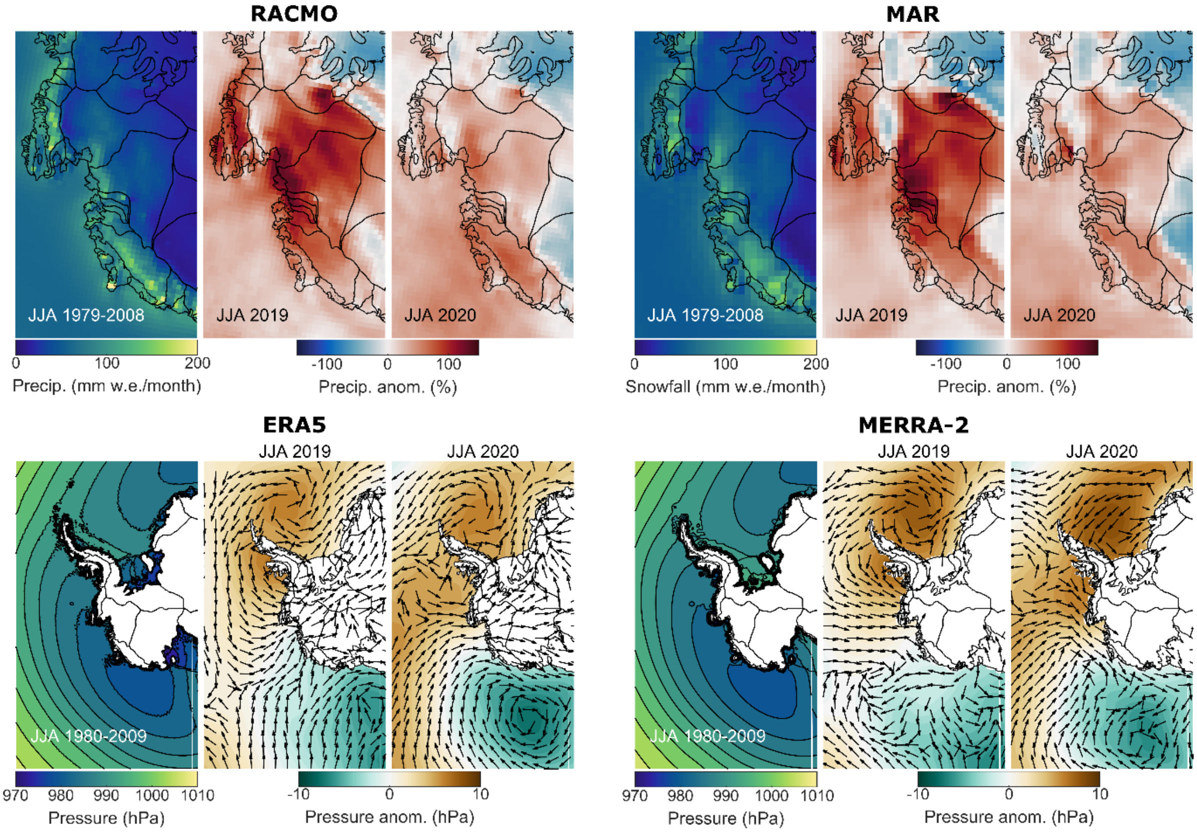

Supplementary Figure 7. Precipitation and pressure anomalies during the 2019 and 2020 winters relative to the winter (JJA) climatological mean in RACMO, MAR, ERA5 and MERRA-2, with the Antarctic Ice Sheet coastline (black line) also shown[1,2].

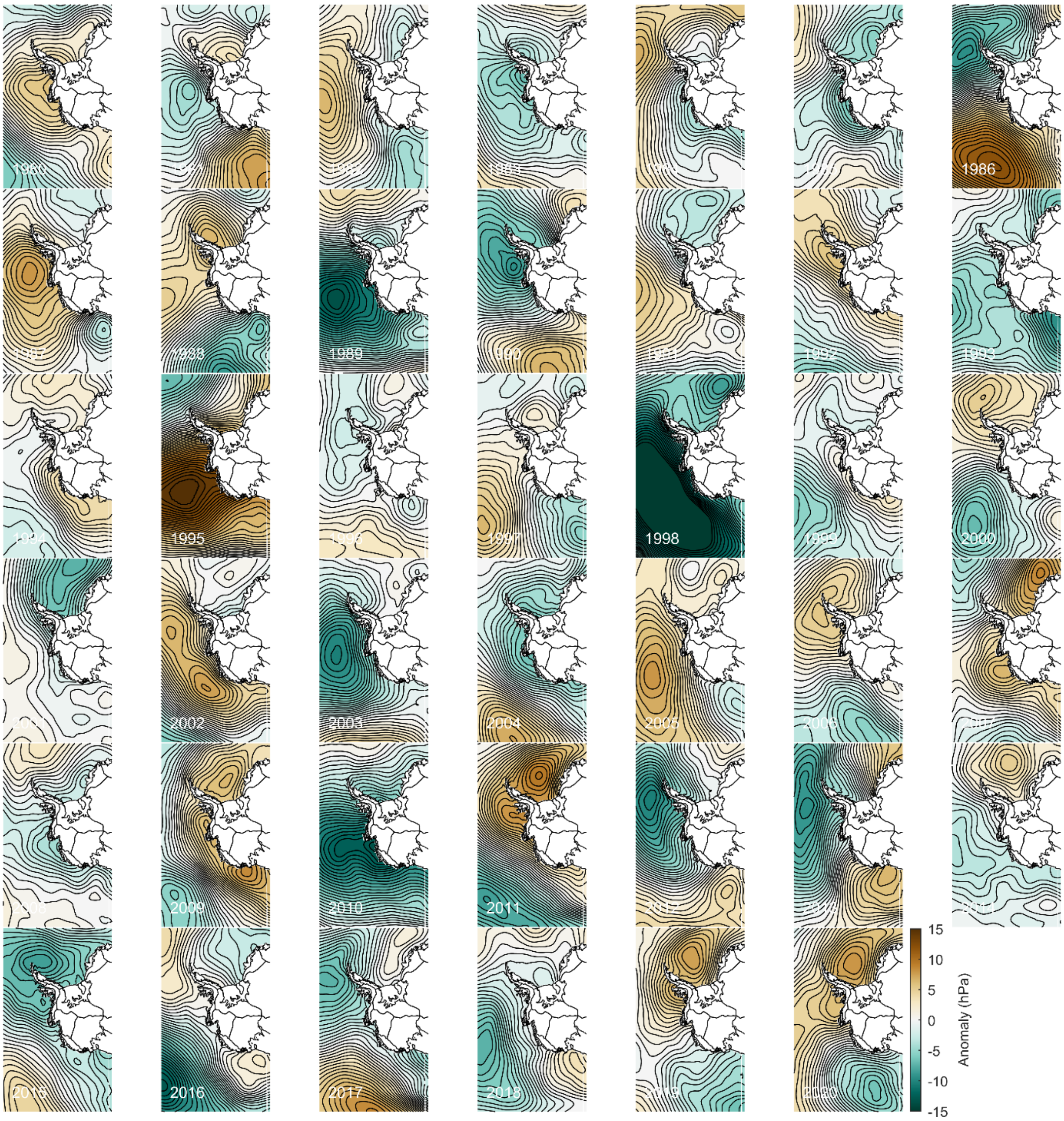

Supplementary Figure 8. Annual June-August (inclusive) surface air pressure anomalies relative to the June-August 1980-2009 mean, from MERRA-2, with the Antarctic Ice Sheet coastline and major drainage basins (black lines) also shown[1,2].

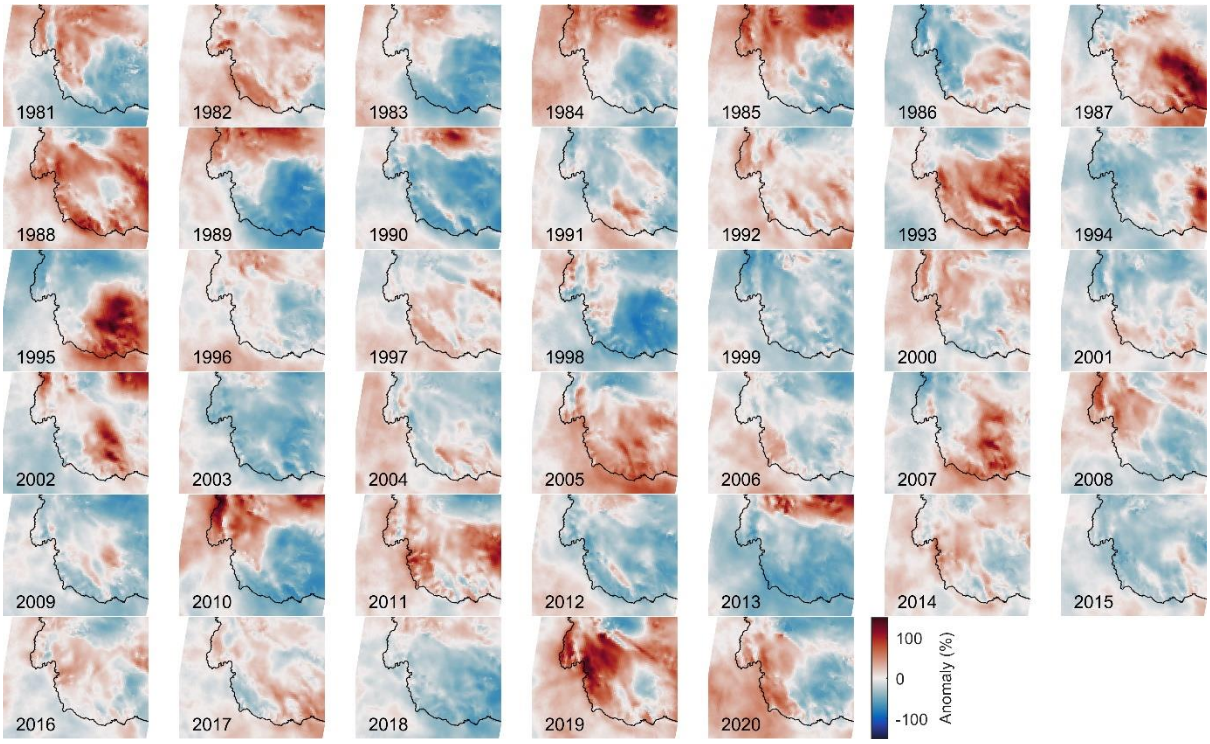

Supplementary Figure 9. Annual June-August (inclusive) precipitation anomalies relative to the June-August 1979-2008 mean, from RACMO2.3p2, with the Antarctic Ice Sheet coastline (black line) also shown[1,2].

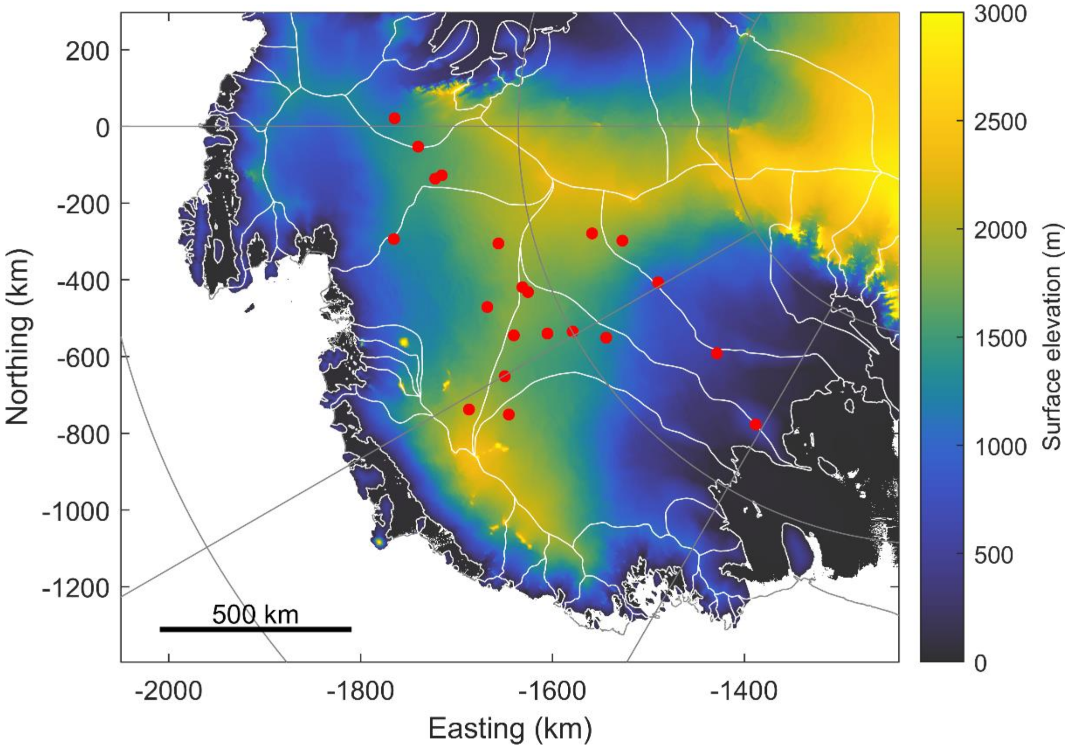

Supplementary Figure 10. Location of ice cores[3] (red dots) plotted in Fig. 6 of the main text. Surface elevations are from the REMA 200m DEM[4]. The Antarctic coastline (grey) 'refined' Antarctic drainage basins (white) are also shown[1,2].

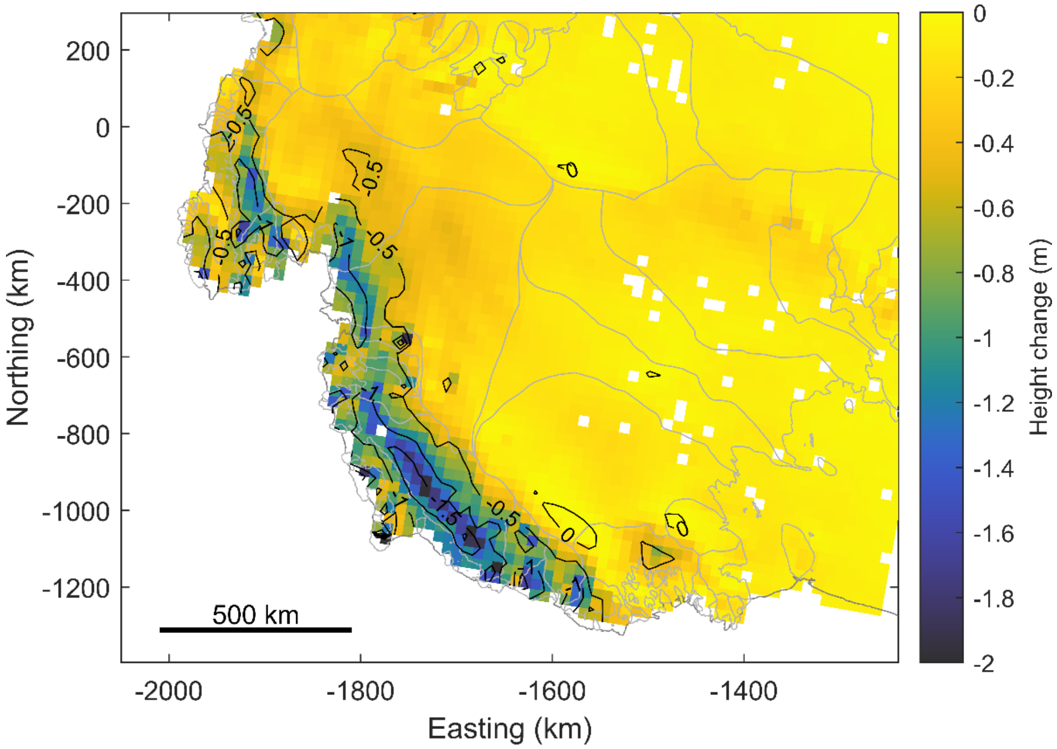

Supplementary Figure 11. Ice-equivalent ice surface height change due to the cumulative surface mass balance anomaly from January 2009 to December 2013 based on RACMO2.3p2. The black contours show the ice equivalent height change at 25 cm increments. The Antarctic coastline (grey) 'refined' Antarctic drainage basins (grey lines) are also shown[1,2].

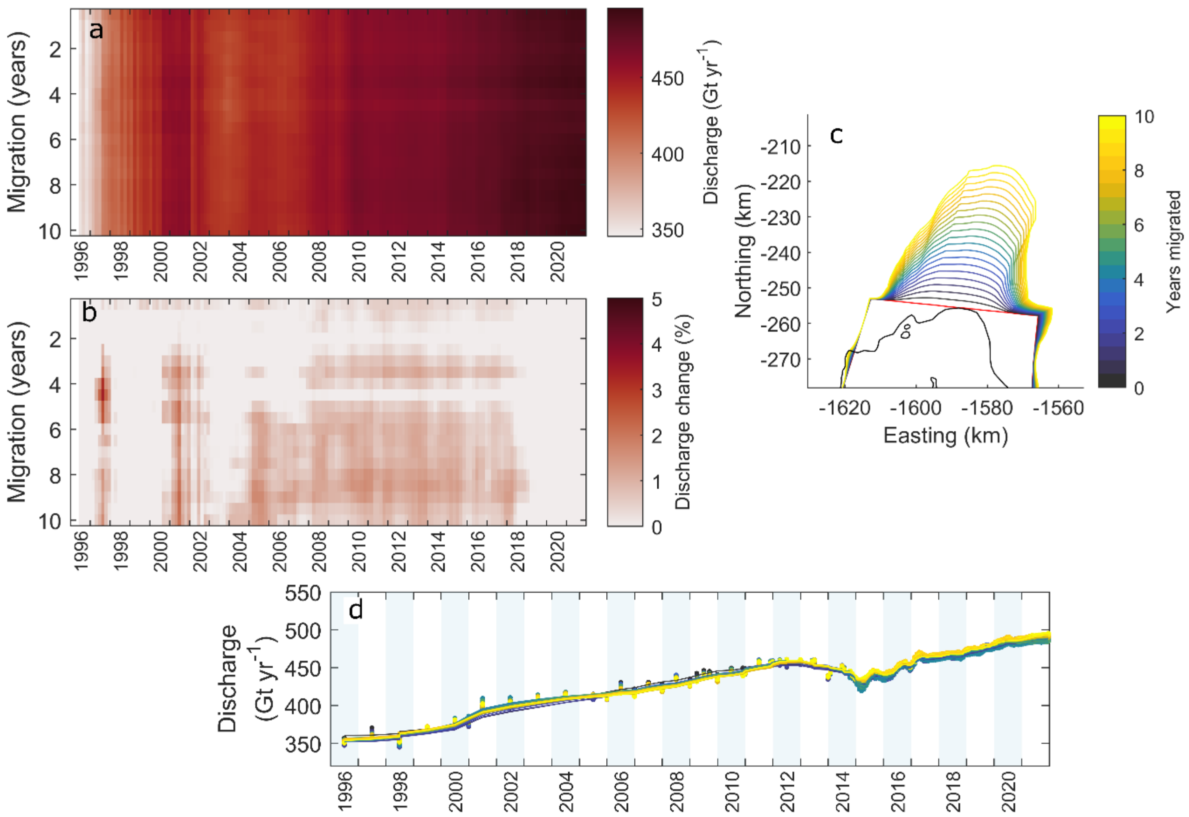

Supplementary Figure 12. Sensitivity of grounding line discharge to flux gate location. (a) Amundsen Sea Embayment (ASE) grounding line discharge through gates migrated in 0.5-year increments for 10 years. (b) Change in discharge through each migrated gate relative to the baseline gate. (c) Example of flux gate locations for Pine Island Glacier, coloured by the number of years of ice flow they have been migrated upstream from the baseline gate (shown in red). (d) Timeseries of ASE discharge through each migrated gate, with observations plotted as filled circles and the 6-point smoothed discharge overlaid as a solid line. Colours in (d) correspond to those in (c). The black line in (c) is the grounding line[1,2].

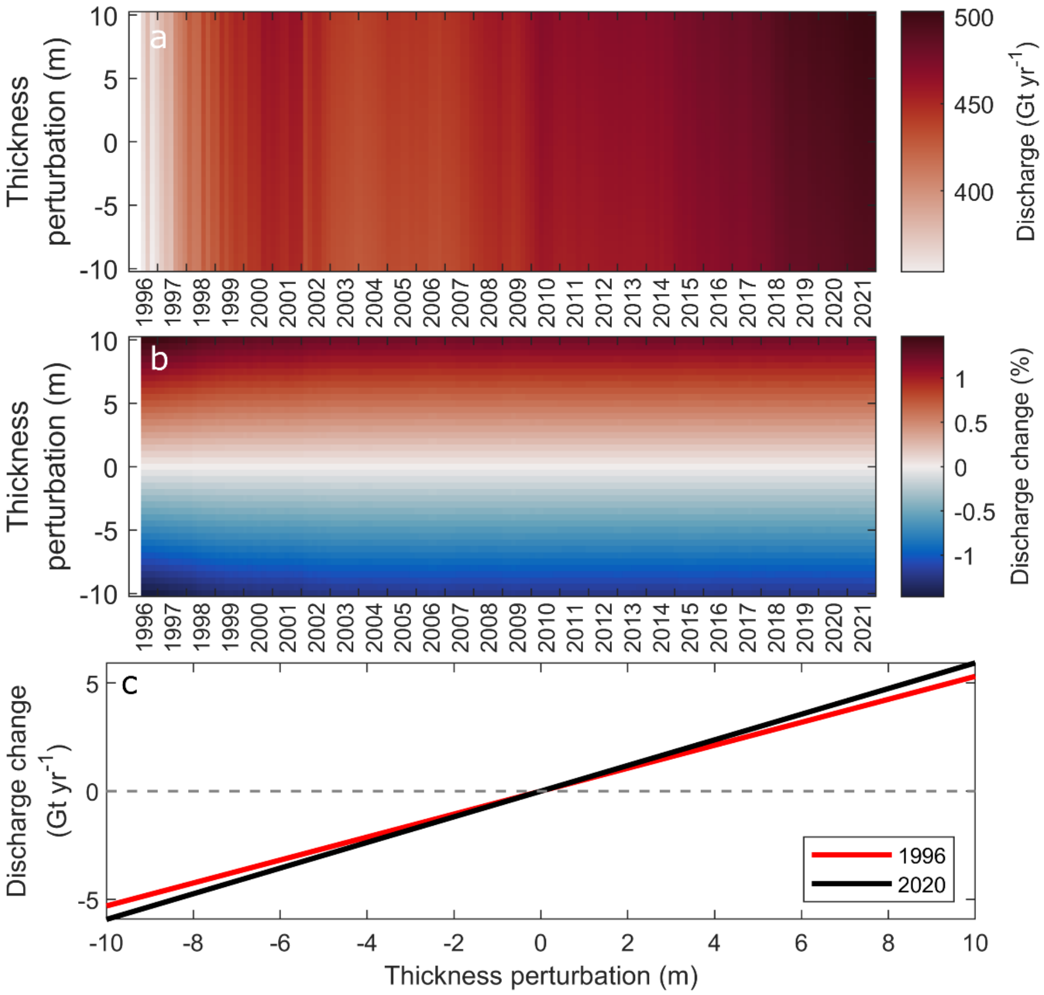

Supplementary Figure 13. Discharge sensitivity to thickness changes. (a) Amundsen Sea Embayment grounding line discharge with different thickness perturbations imposed in addition to the observed thickness changes. (b) Change in discharge with regard to the observed thickness. (c) Absolute change in discharge due to a given thickness perturbation depends on the ice velocity (and therefore time).

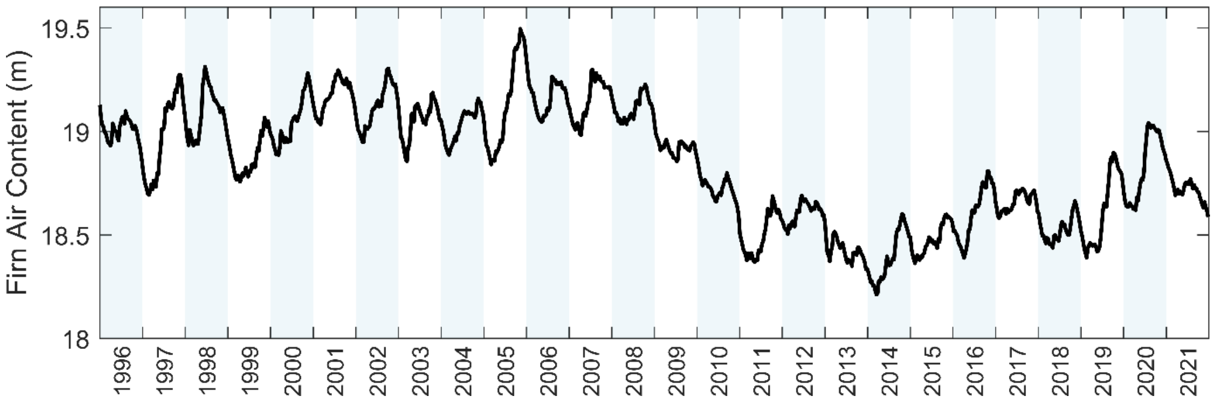

Supplementary Figure 14. Firn air content averaged across all flux gate pixels from the RACMO2.3p2 forced firn densification model. This plot is only illustrative of the applied firn air content correction, which in practice is extracted and applied at each flux gate pixel (i.e. the timeseries for each flux gate pixel is unique).

Supplementary Table 1. Annual (Jan-Dec) grounding line discharge (Gt yr^{-1}), with change relative to 1996 values shown in brackets, for each drainage basin[5].

	Basins 20-22	Basin 22	Basin 21	Basin 20
**1996**	342.7 ± 56.9 (0%)	87.6 ± 12.7 (0%)	130.1 ± 21.6 (0%)	125.6 ± 22.6 (0%)
**1997**	357.1 ± 106.6 (4.2%)	87.9 ± 11.2 (0.4%)	144.6 ± 65.7 (11.2%)	125.2 ± 29.8 (-0.3%)
**1998**	344.9 ± 48.1 (0.7%)	88.6 ± 9.9 (1.1%)	137.4 ± 17.5 (5.6%)	119.6 ± 20.7 (-4.8%)
**1999**	357.3 ± 61.7 (4.3%)	92.5 ± 13.3 (5.7%)	142.5 ± 23.8 (9.6%)	122.8 ± 24.6 (-2.3%)
**2000**	364 ± 59.3 (6.2%)	95.6 ± 14.2 (9.2%)	145.7 ± 19.6 (12%)	123.3 ± 25.5 (-1.9%)
**2001**	381.5 ± 78.7 (11.3%)	102 ± 16.7 (16.4%)	154.4 ± 27.3 (18.7%)	125.7 ± 34.7 (0.1%)
**2002**	387 ± 69.2 (12.9%)	105.8 ± 15.8 (20.8%)	155.6 ± 24.7 (19.6%)	126.2 ± 28.7 (0.5%)
**2003**	393.6 ± 67.1 (14.9%)	109.2 ± 16.7 (24.7%)	157.5 ± 26 (21.1%)	127.5 ± 24.4 (1.5%)
**2004**	404.5 ± 75.6 (18%)	111.6 ± 16.3 (27.4%)	164.3 ± 31.2 (26.3%)	129.2 ± 28.1 (2.9%)
**2005**	400 ± 62.5 (16.7%)	115.2 ± 16.3 (31.6%)	159.3 ± 21.1 (22.5%)	126 ± 25.1 (0.3%)
**2006**	412.3 ± 63.4 (20.3%)	121.1 ± 16.5 (38.3%)	164.8 ± 21.8 (26.7%)	127 ± 25.1 (1.1%)
**2007**	416.3 ± 53.8 (21.5%)	126.1 ± 13 (44%)	166.8 ± 20.1 (28.3%)	124 ± 20.8 (-1.3%)
**2008**	424.4 ± 57.2 (23.9%)	129.3 ± 12.8 (47.6%)	171.5 ± 21 (31.8%)	124.3 ± 23.4 (-1.1%)
**2009**	429.7 ± 66 (25.4%)	130.3 ± 15 (48.8%)	176.1 ± 26.2 (35.4%)	123.9 ± 24.9 (-1.4%)
**2010**	435.8 ± 67.8 (27.2%)	130.5 ± 15.1 (49%)	178.5 ± 24.9 (37.3%)	127.4 ± 27.9 (1.4%)
**2011**	443.6 ± 70.7 (29.4%)	130.7 ± 14.6 (49.2%)	184 ± 26.5 (41.5%)	129.5 ± 29.6 (3.1%)
**2012**	442.3 ± 69.7 (29.1%)	128.6 ± 15.6 (46.8%)	185.4 ± 28.4 (42.6%)	128.8 ± 25.7 (2.5%)
**2013**	438.8 ± 56.7 (28%)	126.1 ± 12.7 (43.9%)	183.1 ± 23.1 (40.8%)	130.2 ± 21 (3.6%)
**2014**	432.3 ± 57.9 (26.2%)	125.1 ± 13.5 (42.8%)	181.3 ± 24.2 (39.4%)	126.5 ± 20.2 (0.7%)
**2015**	428.5 ± 61.6 (25.1%)	123.7 ± 13.8 (41.3%)	182 ± 26.4 (39.9%)	123.4 ± 21.3 (-1.8%)
**2016**	437.9 ± 59.9 (27.8%)	126.1 ± 12.3 (43.9%)	185.9 ± 26.1 (42.9%)	126.5 ± 21.5 (0.7%)
**2017**	452.1 ± 59.1 (31.9%)	128.7 ± 10.4 (46.9%)	191 ± 26 (46.8%)	133 ± 22.7 (5.9%)
**2018**	456 ± 56 (33.1%)	131.8 ± 10.1 (50.4%)	191.3 ± 24.3 (47.1%)	133.5 ± 21.7 (6.2%)
**2019**	465.3 ± 60.5 (35.8%)	137.8 ± 11.4 (57.3%)	193.9 ± 25.9 (49.1%)	134.1 ± 23.2 (6.8%)
**2020**	476.9 ± 61.6 (39.2%)	144.9 ± 11.3 (65.4%)	196.5 ± 26.6 (51.1%)	136.1 ± 23.7 (8.3%)
**2021**	485.6 ± 53.6 (41.7%)	152.6 ± 9 (74.2%)	198 ± 22.3 (52.3%)	135.6 ± 22.4 (7.9%)

Supplementary references

1. Rignot, E., Jacobs, S., Mouginot, J. & Scheuchl, B. Ice-shelf melting around Antarctica. *Science (80-.).* **341**, 266–270 (2013).

2. Mouginot, J., Scheuchl, B. & Rignot, E. MEaSUREs Antarctic Boundaries for IPY 2007-2009 from Satellite Radar, Version 2. *Boulder, Color. USA. NASA Natl. Snow Ice Data Cent. Distrib. Act. Arch. Cent.* (2017). doi:10.5067/AXE4121732AD

3. Thomas, E. R., Hosking, J. S., Tuckwell, R. R., Warren, R. A. & Ludlow, E. C. Twentieth century increase in snowfall in coastal West Antarctica. *Geophys. Res. Lett.* **42**, 9387–9393 (2015).

4. Howat, I. M., Porter, C., Smith, B. E., Noh, M. J. & Morin, P. The reference elevation model of antarctica. *Cryosphere* **13**, 665–674 (2019).

5. Zwally, H. J., Giovinetto, M. B., Beckley, M. A. & Saba, J. L. Antarctic and Greenland Drainage Systems. *GSFC Cryospheric Sci. Lab.* (2012). doi:http://icesat4.gsfc.nasa.gov/cryo_data/ant_grn_drainage_systems.php.
